# Axial Skeletal Location Predicts Poor Outcome in Ewing's Sarcoma: A Single Institution Experience

**DOI:** 10.1155/2011/395180

**Published:** 2011-11-24

**Authors:** Kurt R. Weiss, David J. Biau, Rej Bhumbra, Anthony M. Griffin, Martin E. Blackstein, Peter Chung, Charles Catton, Brian O'Sullivan, Peter C. Ferguson, Jay S. Wunder

**Affiliations:** ^1^Musculoskeletal Oncology Division, Department of Orthopaedic Surgery, University of Pittsburgh, Pittsburgh, PA 15213, USA; ^2^Department of Orthopaedic Surgery, University of British Columbia, Vancouver, BC, Canada V5Z 4E3; ^3^Bone and Soft Tissue Tumour Service, Royal National Orthopaedic Hospital, Middlesex HA7 4LP, UK; ^4^Musculoskeletal Oncology Unit, Division of Orthopaedic Surgery, Mount Sinai Hospital, Toronto, ON, Canada M5G 1X5; ^5^Department of Medicine, Mount Sinai Hospital, Toronto, ON, Canada M5G 1X5; ^6^Department of Radiation Oncology, Princess Margaret Hospital, Toronto, ON, Canada M5G 2M9

## Abstract

*Introduction*. Ewing's sarcomas (EWSs) of bone and soft tissue are neuroectodermal tumors that affect both axial and appendicular locations. We hypothesized that axial location predicted poor outcome in EWS patients. *Materials and Methods*. Sixty-seven patients (57 with bone EWS and 10 with soft tissue EWS) were identified from our database. Thirty-four (51%) had axial EWS and 33 (49%) had appendicular EWS. Statistical analyses identified predictors of poor outcome. *Results and Discussion*. Axial location, large size, metastases at presentation, lack of definitive treatment, and positive surgical margins all correlated with poor outcome in univariate analysis. In multivariate analysis, axial location still predicted poor outcome when adjusted for pretreatment variables. Axial location was not statistically predictive of poor outcome when adjusted for treatment variables. *Conclusions*. Anatomic location has a negative effect on outcome in EWS that cannot be completely explained by pretreatment or treatment factors. Additional studies are required to determine if there is a biologic difference between axial and appendicular EWS.

## 1. Introduction

Ewing's sarcoma (EWS) is a rare malignant neoplasm composed of primitive neuroectodermal cells. It is most commonly found in the skeleton and is the second most common primary tumor of bone. Soft tissue Ewing's sarcoma is much more rare, but is histologically and genetically identical to EWS of bone and is treated similarly [1, 2]. The mainstays of modern treatment for EWS include chemotherapy, surgery, and radiation therapy (XRT). With current treatment strategies, overall survival at 5 years typically ranges between 50 and 70% [1, 3–10]. Most mortality is due to metastatic disease, which occurs in approximately 30% of patients [8, 11, 12].

As EWS carries a guarded prognosis, much effort has been made to discover prognostic factors. Reliable prognostic factors could identify particularly high-risk patients who might benefit from more aggressive treatment approaches and perhaps even investigational agents. Among possible prognostic factors, the presence of metastatic disease at the time of diagnosis, large tumor volume, and poor response to preoperative chemotherapy have been consistently shown to confer a poor prognosis [2, 7–9, 11–16].

Unlike osteosarcoma which favors the femoral and tibial metaphysis, EWS occurs in both axial and appendicular locations. In the Mayo Clinic series, approximately 44% of EWS patients had axial disease [17]. The prognostic importance of anatomic location (i.e., axial versus appendicular) is unclear. Some authors have found anatomic location to be prognostic while others have not [1, 3, 6–9, 11–14, 18–23]. It is difficult to isolate the prognostic effect of anatomic location from other pretreatment variables such as tumor size and the presence of metastatic disease. Preclinical research [24] has suggested that axial location may be associated with a more aggressive biological phenotype than appendicular EWS, but this has not been validated or demonstrated in a clinical setting.

The purpose of this study is to report a single institution experience in the management of patients with bone and soft tissue EWS, and to investigate the importance of anatomic location as a prognostic factor. We hypothesize that axial location confers a worse prognosis than appendicular location. We further hypothesize that axial location independently predicts poor prognosis in EWS and may suggest a biologic difference between axial and appendicular EWS.

## 2. Methods

### 2.1. Patients

After approval from our institutional research ethics board, we searched our sarcoma database for all patients with EWS treated consecutively between 1989 and 2007. A minimum of 2-year followup was required for all patients. Seventy patients were identified. Three had incomplete records and were excluded, leaving 67 patients in the study group. Patients' electronic records, paper charts, and radiographs (when available) were reviewed. Demographic data and details of treatment and clinical course were collected.

Axial bone EWS was defined as disease originating from the spine, sacrum, pelvis, scapula, clavicle, or rib cage. Appendicular bone EWS was defined as disease originating from any bone in the extremities. Axial soft tissue EWS was defined as originating in the head, neck, or torso. Appendicular soft tissue EWS originated from the extremities.

(The scapula and clavicle were included in the axial group because (1) they form via intramembranous ossification, (2) their geometry is clearly different than the long tubular bones of the extremities, and (3) their location overlying the chest wall is more central than the bones of the extremities.)

### 2.2. Staging and Treatment

Staging investigations at diagnosis included computed tomography (CT) scan of the chest and total body bone scan. Some patients received total body gallium scan, magnetic resonance imaging (MRI) of the primary tumor, and bilateral bone marrow aspirations and biopsies. Sixty-six (99%) patients received chemotherapy consisting of vincristine, doxorubicin, and cyclophosphamide (total 5 cycles) alternating with etoposide and ifosfamide (total 5 cycles) for a total of 10 planned treatment cycles.

Definitive local treatment was by en bloc surgical resection whenever possible. XRT was used for lesions that were unresectable, or to treat positive resections margins following surgical management. Patients treated with definitive XRT alone received 5000 centigrays (cGy) in 25 fractions to the entire medullary cavity to cover radiographic bone marrow changes plus a 2cm margin, followed by a boost to 6000–6600 cGy in 30–33 fractions. Similarly, patients with microscopic residual disease postoperatively received 5000 cGy in 25 fractions to the tumor bed plus a 5 cm margin.

### 2.3. Statistical Analysis

The primary outcome measure was overall survival considered from the date of diagnosis to the date of death and estimated with the Kaplan-Meier method. Patients who did not experience death over the study period were censored.

Cox proportional hazard regression models were used to estimate the effect of different variables on overall survival [25]; proportional hazard assumptions were checked using scaled Schoenfeld residuals [26]. The variables considered, besides anatomic location, were tumor size, metastases at diagnosis, treatment with radiation, and surgical margins. In accordance with AJCC guidelines, tumor size was evaluated as greater or less than 8 cm in largest dimension for bone EWS, and greater or less than 5 cm for soft tissue EWS. First, a univariate model was fitted with anatomic location as the sole predictor. If a significant effect of anatomic location was shown, a second multivariate model was fitted with anatomic location and pretreatment variables (size and metastases at diagnosis). This second model would give us some insight as to whether the observed effect of anatomic location could be explained partially by differences in pretreatment variables. Last, a third multivariate model with anatomic location, pretreatment variables, and treatment variables (radiation and surgical margins) was fitted to assess if some effect of anatomic location remained after adjusting for pre-treatment and treatment-related variables. This last model was only fitted in patients who had undergone surgery. Significance was considered for *P* values ≤ 0.05; all tests were two sided.

## 3. Results and Discussion

### 3.1. Results

Of the 67 patients, 41 were male and 26 were female. The mean age at diagnosis was 26.9 years with a range of 12.4–77.9 years. Fifty-seven (85%) patients had bone EWS and 10 (15%) had soft tissue EWS. Thirty-four (51%) patients had axial EWS and 33 (49%) patients had appendicular EWS. The demographic, pre-treatment, and treatment characteristics of the axial and appendicular EWS groups are described in Table 1. Sixty-six (99%) patients received chemotherapy, and 54 (81%) received definitive local treatment with surgery and/or XRT. Margins for the 44 patients who had surgery were negative in 35 patients (80%) and micro- or macroscopically positive in 9 patients (20%). Of all variables, either pre-treatment or treatment, only the status of surgical margins showed a noteworthy difference with axial tumors more likely to be resected with positive margins (6 of 16 compared to 3 of 28; *P* value = 0.053).

**Figure 1:**
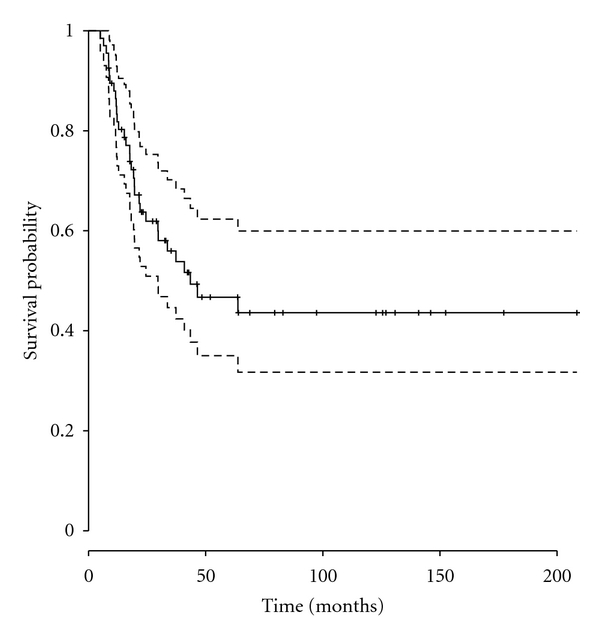
Overall survival, all patients. Figure 1 displays overall survival for all 67 patients. Overall survival was 47% (95% CI = 0.35–0.62) at 5 years and 44% (95% CI = 0.32–0.60) at 10 years.

**Figure 2:**
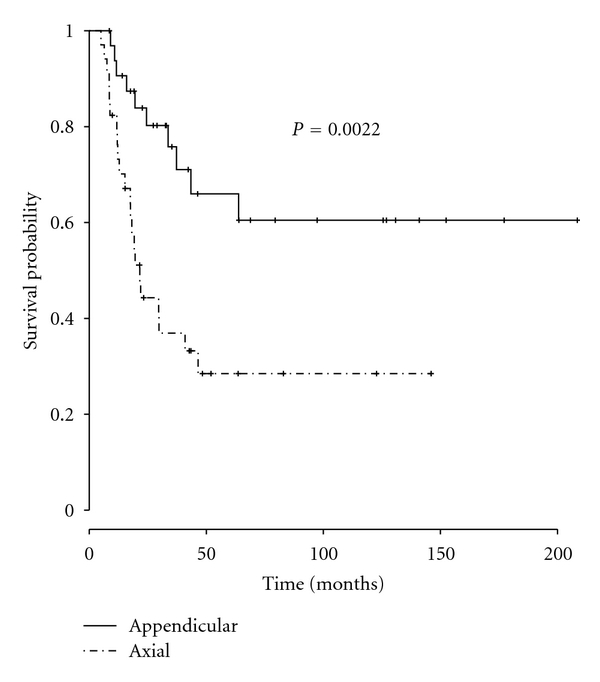
Overall survival, axial versus appendicular. Figure 2 depicts overall survival as a function of anatomic location. Overall survival for the axial group was 29% (95% CI = 0.16–0.52) at 5 years and 10 years. Overall survival was 66% (95% CI = 0.50–0.88) at 5 years and 61% (95% CI = 0.43–0.84) at 10 years. The difference in overall survival between the 2 groups was statistically significant (*P* = 0.002).

Overall survival for the whole group was 64% at 2 years (95% confidence interval: 53% to 77%), 47% at 5 years (95% CI: 35% to 62%), and 44% at 10 years (95% CI: 32% to 60%, Figure 1). Overall survival in patients with axial EWS was 44% at 2 years (95% CI: 30% to 66%), 29% at 5 years, (95% CI: 16% to 52%), and 29% at 10 years (95% CI: 16% to 52%). Comparatively, overall survival in patients with appendicular EWS was 84% at 2 years (95% CI: 72% to 98%), 66% at 5 years, (95% CI: 50% to 88%), and 61% at 10 years (95% CI: 43% to 84%). Overall survival was significantly worse for patients with axial EWS (*P* = 0.002, Figure 2). 

Univariate analysis revealed that axial location (*P* = 0.002), large size (*P* = 0.003), the presence of metastases at diagnosis (*P* < 0.001), and positive margins (*P* = 0.031) all predicted poor overall survival. Treatment with XRT was not significantly associated with worse overall survival (*P* = 0.23, Table 2). In the second model, after adjusting for the effect of pre-treatment variables, axial location remained associated with decreased overall survival with a hazard ratio of 3.11 (95% CI: 1.41 to 6.84; *P* value = 0.005). In the third model, after adjusting for pre-treatment and treatment variables, axial location was associated with decreased overall survival with a hazard ratio of 4.73 (95% CI: 0.87 to 25.7); however this association was not significant (*P* value = 0.072, Table 3).

### 3.2. Discussion

EWS is a member of the “small round blue cell” family of tumors along with rhabdomyosarcoma and lymphoma of bone. It is thought to have a neuroectodermal origin and is characterized by a genetic translocation t(11; 22) in over 95% of cases [27]. We have combined our patients with bone and soft tissue EWS as it has been reported that these two presentations of EWS are similar in terms of genetics, histology, goals of treatment, and prognosis [1, 2].

We found that axial location was predictive of worse overall survival in univariate analysis, and in multivariate analyses when adjusting for pre-treatment variables. Axial skeletal location did not achieve statistical significance when adjusted for pre-treatment and treatment variables (*P* = 0.072) but the hazard ratio was still high (4.73). Argon et al. (*n* = 25), Gupta et al. (*n* = 53), and Hense et al. (*n* = 945) found that anatomic location correlated with poor outcome [3, 11, 13]. Hense et al., in particular, suggested that anatomic location may be more prognostically important than tumor volume. Lee et al. [7] in their series of 725 patients from the California Cancer Registry reported that pelvic site predicted poor overall survival in univariate, but not multivariate analysis. We specifically attempted to isolate the contribution of anatomic location to overall survival by adjusting for pre-treatment (model 2) and treatment (model 3) factors. The fact that the hazard ratio did not vary much between models 1, 2, and 3 suggests that the worse prognosis associated with axial location is explained neither by pre-treatment variables nor treatment variables. However, caution is warranted as the sample size was relatively small and only a limited number of other variables were included in the models.

There were essentially equal proportions of large and small tumors in the axial and appendicular groups by AJCC criteria. It has been widely assumed that the reason for the worse prognosis of axial EWS is because these tumors are larger than those located in the appendicular skeleton. This was not the case in our study population, and our findings suggest that size alone does not explain the difference in prognosis between axial and appendicular EWS.

There was indeed a modicum of treatment heterogeneity. The axial and appendicular groups had different proportions of patients who received surgery (47% versus 85%), who had positive surgical margins (38% versus 10%), and who were treated with definitive XRT (27% versus 3%). It is entirely possible that these treatment differences contributed to the outcome of the axial and appendicular groups.

Hense et al. [11] described an unexplained, intrinsic quality to axial tumors. They postulated that the pelvis posseses “…an additional effect that appears specific to the pelvic site and that was not explained by the variables considered in our statistical analyses. Our study falls short of providing any further clues as to what may constitute this specific feature of pelvic lesions.” We propose that axial EWS may be biologically different from appendicular EWS. For example, in a series of Ewing's sarcoma of the foot and ankle by San-Julian et al., overall survival was 93% [28], which is extraordinarily high. Weiss et al. suggested that axial and appendicular EWS behave differently in an animal model of EWS. Experimental animals were orthotopically implanted with EWS tumor fragments in the leg or the chest wall. Chest wall tumors grew much more reproducibly and aggressively than leg tumors [24]. These data may collectively suggest biologic differences between axial and appendicular EWS that ultimately result in prognostic differences.

## 4. Conclusions

In conclusion, in our study population axial location predicted poor prognosis in EWS. This risk remains after adjusting for pre-treatment and treatment variables. Axial EWS may therefore possess a more aggressive phenotype by virtue of the microenvironmental milieu of the axial skeleton, and the interaction between EWS cells and this microenvironment. Additional clinical and basic scientific investigations will help to prove or disprove this hypothesis.

## Figures and Tables

**Table 1:** Patient Information. Table 1 depicts demographic data, pretreatment characteristics, and treatment characteristics for the axial EWS and appendicular EWS groups.

	Axial EWS (*n* = 34, 51%)	Appendicular EWS (*n* = 33, 49%)	Total (*n* = 67)	*P* value
Pretreatment Factors				
Median Age	25.1	28.7	26.9	0.23
No. male	21 (62%)	20 (61%)	41 (61%)	0.30
No. female	13 (38%)	13 (39%)	26 (39%)	—
No. bone	31 (91%)	26 (79%)	57 (85%)	0.19
No. soft tissue (ST)	3 (9%)	7 (21%)	10 (15%)	—
No. with metastases at diagnosis	10 (29%)	5 (15%)	15 (22%)	0.33
No. bone lesions over 8 cm	21 (62%)	17 (52%)	38 (57%)	0.9
No. bone lesions under 8 cm	10 (29%)	9 (27%)	19 (28%)	—
No. ST lesions over 5 cm	1 (3%)	4 (12%)	5 (7%)	1.0
No. ST lesions under 5 cm	2 (6%)	3 (9%)	5 (7%)	—

Treatment Factors				
No. receiving definitive treatment	25 (74%)	29 (88%)	54 (81%)	0.22
No. receiving definitive surgery	16 (47%)	28 (85%)	44 (66%)	0.0018
No. receiving definitive XRT	9 (26%)	1 (3%)	10 (15%)	0.013
No. not receiving surgery or XRT	9 (26%)	4 (12%)	13 (19%)	0.22
No. receiving adjuvant XRT	8 (24%)	2 (6%)	10 (15%)	0.045

Outcome Parameters				
No. positive margins	6 (18%)	3 (9%)	9 (13%)	0.0031
No. local recurrences	5 (15%)	0 (0%)	5 (7%)	0.053
No. ANED	10 (29%)	22 (67%)	32 (48%)	
No. AWED	2 (6%)	1 (3%)	3 (4%)	
No. DOD	22 (65%)	10 (30%)	32 (48%)	
% 5-year OS	29%	66%		0.002

**Table 2:** Effects of pre-treatment and treatment variables in univariable Cox proportional hazard models.

Variable/model	Univariate model	
	HR [95% CI]	*P* value
Site (axial)	3.07 [1.44–6.52]	0.0022
Size (large)	3.8 [1.46–9.9]	0.0034
Metastasis (yes)	4.1 [1.99–8.43]	<0.001
Radiation (yes)	1.53 [0.76–3.11]	0.23
Surgical margins (positive)	3.3 [1.04–10.43]	0.031

HR (hazard ratio); 95% CI (95% confidence interval).

**Table 3:** Effect of anatomic location in univariable (model 1), multivariable analysis when adjusting for pre-treatment variables (model 2), and when adjusting for pre-treatment and treatment variables (model 3).

Variable/model	Model 1		Model 2		Model 3	
	HR [95% CI]	*P* value	HR [95% CI]	*P* value	HR [95% CI]	*P* value
Site (axial)	3.07 [1.44–6.52]	0.0022	3.11 [1.41–6.84]	0.0048	4.73 [0.87–25.74]	0.072
Size (large)	—	—	3.85 [1.44–10.32]	0.0072	4.35 [0.78–24.34]	0.094
Metastasis (yes)	—	—	2.9 [1.37–6.13]	0.0055	2.99 [0.31–5.01]	0.34
Radiation (yes)	—	—	—	—	0.79 [0.13–5.01]	0.81
Surgical margins (positive)	—	—	—	—	2.47 [0.63–9.63]	0.19

HR (hazard ratio); 95% CI (95% confidence interval).
